# Herpes simplex virus 1 UL41 protein abrogates the antiviral activity of hZAP by degrading its mRNA

**DOI:** 10.1186/s12985-015-0433-y

**Published:** 2015-12-01

**Authors:** Chenhe Su, Jie Zhang, Chunfu Zheng

**Affiliations:** Institutes of Biology and Medical Sciences, Soochow University, Suzhou, 215123 China; Wuhan Institute of Virology, Chinese Academy of Sciences, Wuhan, 430071 China; Adjunct professor, Department of Microbiology, Immunology and Infectious Deseases, University of Calgary, Calgary, AB T2N 4 N1 Canada

**Keywords:** HSV-1, hZAP, UL41, Immune evasion

## Abstract

**Background:**

The zinc finger antiviral protein (ZAP) is a host restriction factor that inhibits the replication of various viruses by degradation of certain viral mRNA. However, previous study demonstrated that ectopic expression of rat ZAP did not suppress the replication of herpes simplex virus type 1 (HSV-1), an archetypal member of the alphaherpesvirus subfamily, and the molecular mechanism underneath is still illusive.

**Results:**

Human ZAP (hZAP) does not suppress the replication of herpes simplex virus 1, and HSV-1 UL41 protein was identified as an antagonist of hZAP by degrading its mRNA. Infection of wild-type (WT), but not UL41-null mutant (R2621) virus, diminished the accumulation of hZAP to abrogate its antiviral activity. Moreover, ectopic expression of hZAP inhibited the replication of R2621 but not WT HSV-1.

**Conclusion:**

HSV-1 UL41 was shown for the first time to evade the antiviral function of hZAP via its RNase activity.

## Background

Herpes simplex virus 1 (HSV-1) is the archetypal member of the alphaherpesvirus subfamily with a large genome encoding over 80 viral proteins. UL41 is a HSV-1 tegument protein, and also a minor structural component of HSV-1 virions [1, 2], and it can degrade host mRNAs by cutting them at preferred site [3–5]. Upon HSV-1 infection, UL41 polypeptides enter the cell as components of infecting virions and contributes to an overall decrease in host protein synthesis [6]. By shortening the half-life of mRNAs, UL41 helps to redirect the cell from host to viral gene expression, and facilitates the sequential expression of different classes of viral genes [7]. Therefore, HSV-1 evades host responses to infection and diverts the resources of the cells to viral macromolecular synthesis.

The ZAP (zinc finger antiviral protein) was originally discovered in rat as an antiretroviral factor [8]. The viruses that contain ZAP-responsive element (ZRE) in their viral mRNAs are sensitive to ZAP [9]. Human ZAP (hZAP) exists in two isoforms, hZAPL and hZAPS respectively [10]. Both hZAP isoforms have antiviral effects against several RNA viruses [11, 12], while only ZAPS, not ZAPL was up-regulated under IFN treatment [13, 14].

HSV-1 UL41 is an endoribonuclease with the substrate specificity of RNase A [15]. ZAP could not inhibit HSV-1 infection [16], and the molecular mechanism underneath is still illusive. In this study, we show for the first time that HSV-1 UL41 abrogate s hZAP’s antiviral activity by degrading hZAP mRNA.

## Results

### HSV-1 UL41 protein antagonized the antiviral activity of hZAP

**Fig. 1:**
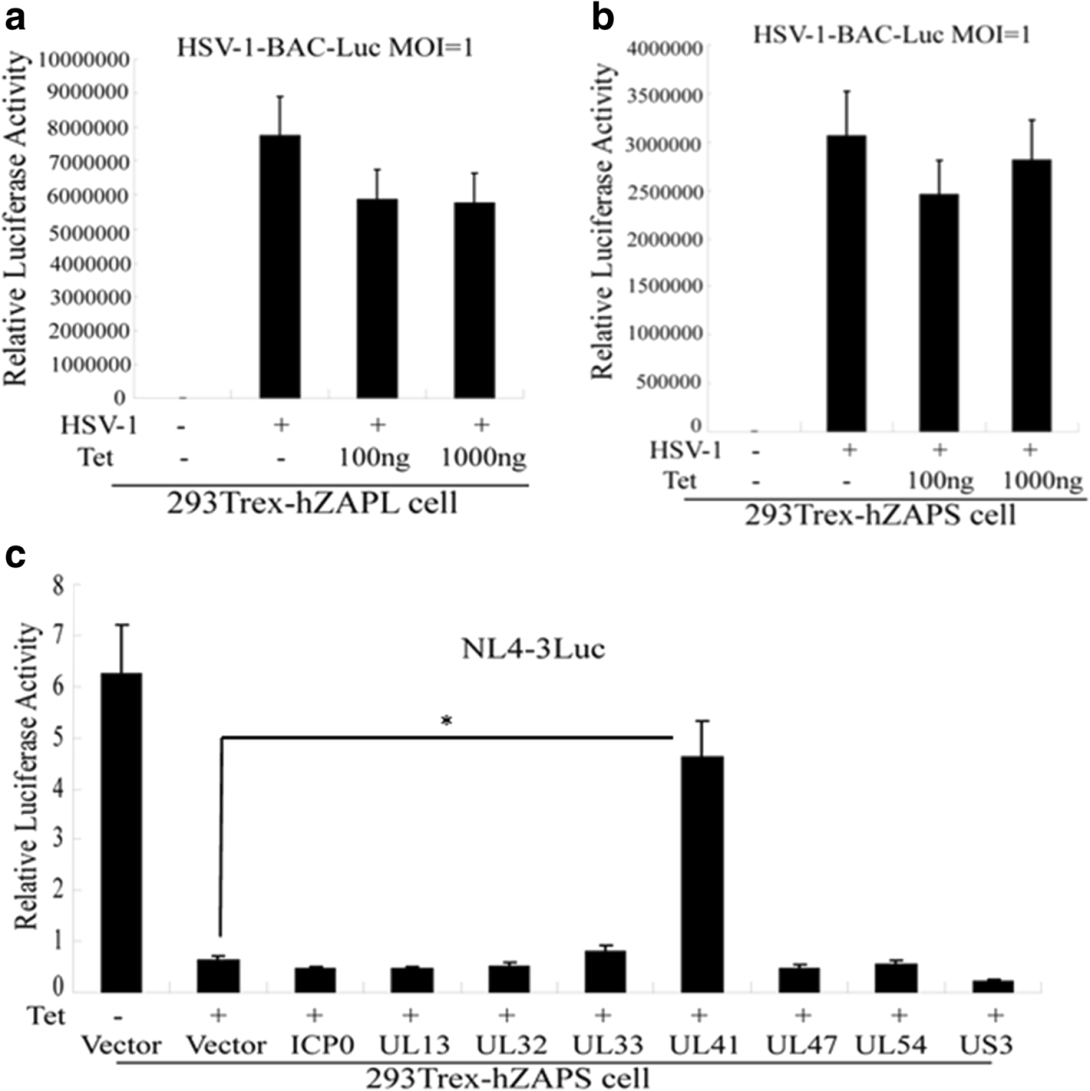
HSV-1 UL41 protein abrogated the antiviral activity of hZAP. **a** and **b** 293Trex-hZAPL cells and 293Trex-hZAPS cells were infected with HSV-1-BAC-Luc at an MOI of 1. Cells were treated or mock treated with tetracycline (100 ng/mL or 1000 ng/mL) 2 h post-infection to induce hZAP expression. The cells were lysed, and luciferase activities were measured at 36 h after infection. Fold inhibition was calculated as the ratio of the luciferase activity in mock-treated cells to that in tetracycline-treated cells. **c** 293Trex-hZAPS cells were transfected with 500 ng of NL4-3-Luc reporter plasmid, together with *Renilla* luciferase plasmid pRL-TK (50 ng) and pEYFP-N1 control vector or plasmids encoding the indicated viral proteins (1000 ng). At 6 h post-transfection, cells were treated with or without tetracycline (1000 ng/mL) to induce hZAPS expression and were incubated for additional 36 h, followed by cell lysis. The luciferase activity was determined by a dual-luciferase assay. Data shown are means ± SD from three separate experiments. (*P < 0.05)

Previous study showed that ectopic expression of rat ZAP did not affect HSV-1 infection [16]. To investigate whether hZAP could inhibit HSV-1 infection, 293TRex-hZAPL/ZAPS cells, which expressed ZAPL/ZAPS in a tetracycline (Tet)-inducible manner, were infected with HSV-1-BAC-Luc at an MOI of 0.1, 1 or 10 and then mock-treated or treated with 100 ng/mL or 1000 ng/mL Tet [12, 17]. Luciferase activity was measured to determine the replication of viruses. As a result, different MOI of HSV-1 replicated similarly in mock and hZAPL- or hZAPS-expressing cells (Fig. 1a, b and data not shown). Viral plaque assay was also conducted and similar result was obtained (data not shown). These results demonstrate that hZAP does not inhibit HSV-1 infection.

To determine whether any of HSV-1 proteins could dampen the antiviral activity of hZAP, a high-throughput screening assay was applied to test all HSV-1 encoded proteins [18]. Tetracycline treatment resulted in strong inhibition of NL4-3-luc expression, which contains the most of the sequence of the HIV-1 genome [12]. In contrast, ectopic expression of UL41 significantly promoted the expression of NL4-3-luc under tetracycline treatment, but not the other proteins of HSV-1 (Fig. 1c). Ectopic expression of UL41 could also facilitate VSV-G-pseudotyped NL4-3-luc virus infection in 293Trex-hZAPL cells or 293Trex-hZAPS cells in a dose dependent manner (data not shown). Taken together, these results demonstrate that UL41 antagonizes the antiviral activity of hZAP.

### HSV-1 UL41 protein downregulated the expression of hZAP

**Fig. 2:**
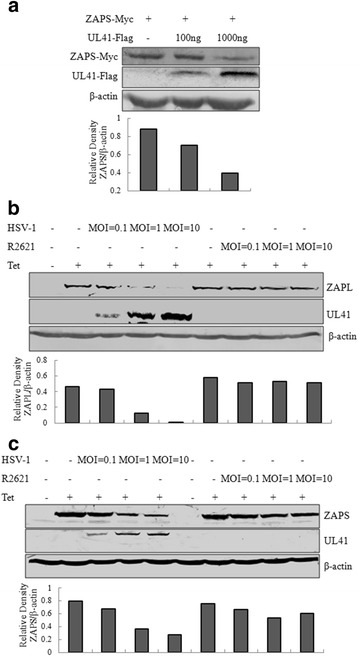
HSV-1 UL41 protein inhibits the expression of hZAP. **a** HEK 293 T cells were co-transfected with hZAPS-Myc plasmid along with increasing amounts of UL41-Flag plasmid. 24 h after transfection, cells were lysed and the samples were then subjected to WB analysis. The lower panel presented relative density analysis of hZAPS. **b** and **c** 293Trex-hZAPL cells and 293Trex-hZAPS cells were infected with WT HSV-1 or R2621 at an MOI of 0.1, 1 or 10, respectively. At 2 h post-infection, cells were mock treated or treated with tetracycline (1000 ng/mL). Cells were lysed and the samples were subjected to WB analysis 36 h post-infection. The relative density analysis of hZAPL and hZAPS were under the WB results, respectively. One representative of three independent experiments was shown

The aforementioned results demonstrated that UL41 was an antagonist of hZAP protein. In order to clarify the molecular mechanism of UL41 to abrogate hZAP antiviral activity, HEK 293 T cells were co-transfected with hZAPS-Myc plasmid and increasing amounts of UL41-Flag plasmids. As a result, ectopic expression of UL41-Flag reduced the abundance of hZAPS in a dose dependent manner (Fig. 2a).

To investigate whether hZAP was downregulated during HSV-1 infection, 293Trex-hZAPL cells or 293Trex-hZAPs cells were infected with WT HSV-1 or R2621 at an MOI of 0.1, 1 and 10. As a result, hZAPL and hZAPS were significantly decreased during WT HSV-1 infection at an MOI of 10, while R2621 did not affect the expression of hZAPL and hZAPS (Fig. 2b, c). Taken together, all these results suggest that UL41 reduced the expression of hZAP.

### HSV-1 UL41 protein promoted the degradation of hZAP mRNA

**Fig. 3:**
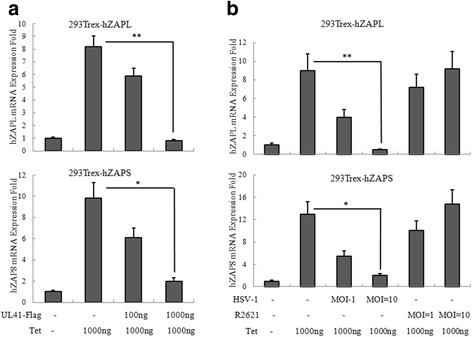
HSV-1 UL41 protein promotes hZAP mRNA degradation. **a** 293Trex-hZAPL or 293Trex-hZAPS cells were transfected with pCMV-Flag control vector or increasing amount of UL41-Flag plasmid. At 6 h post-transfection, cells were mock treated or treated with tetracycline (1000 ng/mL) to induce hZAPL or hZAPS expression. Quantitative RT-PCR analysis was then performed to detect the mRNA level of hZAPL or hZAPS. **b** 293Trex-hZAPL cells or 293Trex-hZAPS cells were infected with WT HSV-1 or R2621 at an MOI of 1 or 10, respectively. At 2 h post-infection, cells were mock treated or treated with tetracycline (1000 ng/mL). Quantitative RT-PCR analysis was then performed to detect the mRNA level of hZAPL or hZAPS. One representative of three independent experiments was shown. (*P < 0.05, **P < 0.01)

UL41 was an endoribonuclease with its substrate specificity similar to that of RNase A [15]. Therefore, we hypothesize that UL41 decreases hZAP expression via its RNase activity to degrade hZAP mRNA. To test this hypothesis, UL41-Flag plasmid was transfected into 293Trex-hZAPL and 293Trex-hZAPS cells, and cells were treated with tetracycline (1000 ng/mL) 6 h post-transfection. As a result, ectopic expression of UL41-Flag downregulated the abundance hZAPL and hZAPS mRNA in a dose dependent manner (Fig. 3a).

To investigate whether the mRNA of hZAP was downregulated during viral infection, 293Trex-hZAPL and 293Trex-hZAPs cells were infected with WT HSV-1 or R2621 at an MOI of 1 and 10. As a result, the mRNA levels of hZAPL and hZAPS were significantly decreased during WT HSV-1 infection at an MOI of 10 (Fig. 3b). However, R2621 failed to degrade hZAPL and hZAPS mRNA (Fig. 3b), indicating that UL41 was responsible for the degradation of hZAPL and hZAPS mRNA. Taken together, these results demonstrated that UL41 dampened the antiviral activity of hZAP by degradation its mRNA.

### Ectopic expression of hZAP inhibited UL41-null mutant HSV-1 infection

**Fig. 4:**
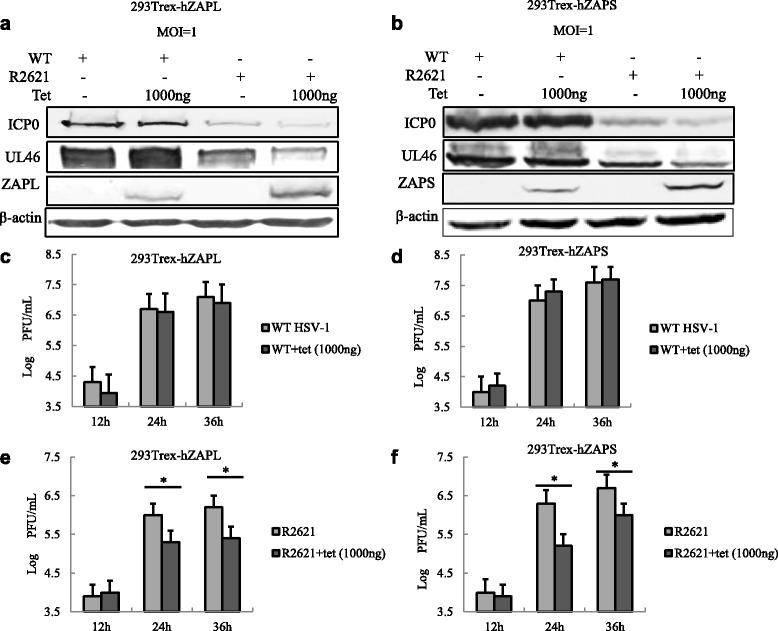
Expression of hZAP inhibits UL41-null mutant HSV-1 R2621 infection. **a** and **b** 293Trex-hZAPL cells or 293Trex-hZAPS cells were infected with WT HSV-1 or R2621 at an MOI of 1, respectively. At 2 h post-infection, cells were mock treated or treated with tetracycline (1000 ng/mL). At 36 h post-infection, cells were lysed and subjected to WB analysis with the indicated Ab. **c** and **d** 293Trex-hZAPL cells or 293Trex-hZAPS cells were infected with WT HSV-1. After 2 h post-infection, cells were treated with or without Tet (1000 ng/mL) to induce hZAPL or hZAPS expression. Viral growth curves were generated by traditional plaque assays at the indicated time points. **e** and **f** 293Trex-hZAPL cells or 293Trex-hZAPS cells were infected with R2621 virus. After 2 h post-infection, cells were treated with or without Tet (1000 ng/mL) to induce hZAPL or hZAPS expression. Viral growth curves were generated by traditional plaque assays at the indicated time points. One representative of three independent experiments was shown. (*P < 0.05)

Our previous results suggested that HSV-1 UL41 protein reduced the antiviral activity of hZAP by degradating its mRNA. However, the UL41-null virus R2621 exerts no such function, which leads us to wonder whether hZAP could inhibit R2621 infection. Thus, 293Trex-hZAPL cells or 293Trex-hZAPS cells were infected with WT HSV-1 or R2621 at an MOI of 1. At 2 h post-infection, the cells were treated or mock treated with tetracycline (1000 ng/mL). Western blot (WB) was performed to analyze the replication of these viruses 36 h post infection. As a result, the expression levels of ICP0 and UL46 in WT HSV-1 infected hZAPS and hZAPL-expression cells were similar to the WT HSV-1 infected mock treated cells. While the expression levels of ICP0 and UL46 in R2621 infected cells were inhibited by hZAPS or hZAPL expression compared to the R2621 infected mock treated cells (Fig. 4a, b). WT HSV-1 or R2621 viral growth curves were also examined through viral plague assays. As a result, ectopic expression of hZAPL or hZAPS inhibited UL41-null HSV-1 replication, but not WT HSV-1 replication (Fig. 4c-f). Taken together, these results indicate UL41 promotes HSV-1 infection by antagonizing the antiviral activity of hZAP.

## Discussion

ZAP is an intrinsic host antiviral factor, which has been reported to not only inhibit the infection of a variety of RNA viruses [8, 11, 12, 16, 19], but also several DNA viruses [9, 14]. ZAP specifically inhibits the replication of many viruses by preventing the accumulation of viral mRNAs in the cytoplasm [20]. However, previous study showed that HSV-1 could resist the antiviral effect of ZAP [16]. In this study, we showed that hZAP does not suppress HSV-1 replication using the recombinant HSV-1 BAC-Luc, which behaved indistinguishably from the wild-type HSV-1 but could be easily quantified in vitro due to its luciferase activity [17]. Furthermore, HSV-1 UL41 protein was demonstrated for the first time to degrade hZAP mRNA and dampen the antiviral activity of hZAP.

UL41 triggers degradation of host mRNAs and rapid shutoff of host cell protein synthesis [21]. By shortening the half-life of mRNAs, UL41 helps redirect the cell from host to viral gene expression, and facilitates the sequential expression of different classes of viral genes [7]. And three amino acid residues E192, D194, and D195 of UL41 are essential for the nuclease activity [15]. UL41 mutants reportedly display a 5- to 10-fold reduction in virus yield in tissue culture infection [1, 7, 22]. Previous studies demonstrated that UL41 could inhibit IFN-mediated antiviral response via multiple mechanisms [23]. Moreover, UL41 was reported to reduce the expression of innate immune sensors, such as TLR2, TLR3, RIG-I and Mda-5 [24]. and block IFN-γ signaling by reducing the expression of the IFN-gamma receptor alpha chain (IFNGR1) [25]. Here, we found that UL41 could degrade hZAP mRNA, and that may facilitate the replication of HSV-1.

ZAP is an interferon-inducible gene and exhibits intrinsic antiviral activity [26, 27]. Previous study demonstrated that MHV-68 infection up-regulates ZAP expression. ZAP inhibits the expression of MHV-68 ORF64, but MHV-68 RTA antagonizes the antiviral activity of ZAP [28]. In this study, we demonstrate that HSV-1 infection down-regulates hZAP expression, and HSV-1 UL41 degrades hZAP mRNA. These findings will lead to a better understanding of the mechanisms employed by HSV-1 UL41 to dampen host antiviral signaling and develop the therapeutic interventions to modulate HSV-1 pathogenesis.

## Conclusions

In summary, we have shown here for the first time that HSV-1 UL41 abrogate s the antiviral activity of hZAP by targeting its mRNA for degradation, and consequently inhibiting the expression of hZAP. These findings will lead to a better understanding of the mechanisms employed by HSV-1 UL41 to dampen host antiviral signaling and develop the therapeutic interventions to modulate HSV-1 pathogenesis.

## Materials and methods

### Cells, viruses and antibodies

HEK 293 T cells, Vero cells, 293Trex-hZAPL and 293Trex-hZAPS cells were cultured in Dulbecco’s modified Eagle medium (DMEM) (Gibco-BRL) supplemented with 10 % fetal bovine serum (FBS) and 100 U/ml of penicillin and streptomycin. WT HSV-1 (strain F) and HSV-1-BAC-Luc (expressing firefly luciferase) were multiplied, titered and purified as previously described [17]. The UL41-null mutant virus (R2621) was propagated and titered as described previously [29].

Mouse anti-Myc and anti-Flag monoclonal antibodies (MAbs), Rabbit polyclonal anti-ZAPL and Rabbit polyclonal anti-ZAPS, Mouse anti-β-actin MAb were purchased from ABmart (Shanghai, China), Proteintech (Wuhan, China), Santa Cruz Biotechnology (Santa Cruz, CA), respectively. Rabbit polyclonal anti-ICP0, UL42 and UL46 were purchased from GL Biochem Ltd. (Shanghai, China), and Rabbit polyclonal anti-UL41 was kindly provided by Dr. Roizman. Tetracycline (Tet) was purchased from Biovision (San Francisco, CA).

### Transfection, infection and dual-luciferase reporter (DLR) assay

HEK 293 T cells were transfected by standard calcium phosphate precipitation whereas hZAP-expressing cells were transfected using Lipofectamine 2000 (Invitrogen). VSV-G-pseudotyped NL4-3-luc virus were produced by transiently co-transfecting HEK 293 T cells with pVSV-G plasmid and pNL4-3-luc. 293Trex-hZAPS cells were co-transfected with reporter plasmid pNL4-3-Luc and internal control plasmid pRL-TK, with or without expression plasmids. At 6 h post-transfection, cells were mock treated or treated with Tetracycline (1000 ng/mL) for 36 h, and then luciferase assays were performed as previously described [30].

### RNA isolation, quantitative RT-PCR

Total RNA was extracted using Trizol (Invitrogen, California) according to the manufacturer’s manual. Samples were digested with DNase I and subjected to reverse transcription previously described [30]. The cDNA was used as template for quantitative PCR (qPCR) to investigate the expression of hZAP. qPCR was performed according to the manufacturer’s instructions (SYBR Premix Ex Taq, Takara, Japan), and 18S rRNA was used as internal reference.

### Statistical Analysis

The data were evaluated with the Student’s t-test and *p* < 0.05 were considered statistically significant.

## Abbreviations

DLR: dual-luciferase reporter assay
HSV-1: herpes simplex virus 1
hZAP: human zinc finger antiviral protein
MOI: multiplicity of infection
R2621: UL41-null mutant virus
rZAP: rat zinc finger antiviral protein
Tet: tetracycline
VSVG: vesicular stomatitis virus G protein
WT: wild-type
ZAP: zinc finger antiviral protein
ZRE: ZAP-responsive element
